# Measurements using the alkaline comet assay predict bladder cancer cell radiosensitivity

**DOI:** 10.1038/sj.bjc.6601333

**Published:** 2003-12-09

**Authors:** M A L Moneef, B T Sherwood, K J Bowman, R C Kockelbergh, R P Symonds, W P Steward, J K Mellon, G D D Jones

**Affiliations:** 1 Department of Cancer Studies and Molecular Medicine, Hodgkin Building, University of Leicester, Lancaster Road, PO Box 138, Leicester LE1 9HN, UK; 2 University Division of Urology, Leicester General Hospital, Gwendolen Road, Leicester LE5 4PW, UK; 3 University Department of Cancer Studies and Molecular Medicine, Osborne Building, Leicester Royal Infirmary, Leicester LE1 5WW, UK

**Keywords:** bladder cancer, comet assay, radiosensitivity, DNA damage, radiotherapy

## Abstract

In the UK, the two main treatments of invasive bladder cancer are radiotherapy or cystectomy. However, ∼50% of patients undergoing radiotherapy fail to respond. If tumour radiosensitivity could be predicted in advance, it may be possible to improve control rates significantly by selecting for radiotherapy those patients whose tumours are radiosensitive. Additionally, patients who would benefit from surgery would be identified earlier. The alkaline comet assay (ACA) is a sensitive method for the detection of DNA strand break damage in cells. In the present study, using six bladder cancer cell lines of differing radiosensitivities, cell survival was compared to the manifestation of radiogenic DNA damage as assessed by ACA. For all the cell lines, the extent of comet formation strongly correlates with cell killing (*R*^2^>0.96), with a greater response being noted in radiosensitive cells. In repair studies, measures of residual damage correlate with survival fraction at 2 Gy (*R*^2^>0.96), but for only five of the cell lines. Finally, cells from human bladder tumour biopsies reveal a wide range of predicted radiosensitivies as determined by ACA. Overall, these studies demonstrate ACA to be a good predictive measure of bladder cancer cell radiosensitivity at low dose, with potential clinical application.

In the UK, bladder cancer is a common urological malignancy, affecting more than 12 500 individuals each year and causing nearly 5000 deaths per annum (CRC, 2001). In all, 90% of bladder cancers are transitional cell carcinomas (TCCs) and ∼30% of bladder tumours are muscle invasive at presentation, a feature that is associated with significant risk of metastasis (30–60%). Patients with organ-confined, muscle-invasive tumours, (T2/3/4a) who are deemed fit, are considered for potentially curative treatment in the form of radical surgery (cystectomy) or radiotherapy. Radical cystectomy (removal of the bladder and urinary diversion) is associated with significant morbidity and a mortality rate of ∼2% (Skinner *et al*, 1988; Studer *et al*, 1995). Furthermore, the necessary creation of an abdominal wall stoma (ileal conduit) may impact on quality of life, and erectile dysfunction is virtually universal. The outcome following radical cystectomy varies with tumour stage, with an overall 5-year survival of ∼40%.

Radical radiotherapy (RT) is the mainstay of bladder-sparing treatment regimens. It avoids the trauma of major surgery and is considered a primary treatment for patients deemed unfit for surgery. However, RT is itself associated with dose-related complications arising from bowel and bladder being included in the radiation field. Furthermore, while for ∼50% of patients radical RT results in effective local tumour control and acceptable bladder function (Quilty *et al*, 1986; Jenkins *et al*, 1989), the remaining patients suffer local recurrence. For this latter group, the decision to treat with RT is disadvantageous, as these patients have been unnecessarily exposed to additional risks of ionising radiation. In addition, the time taken to recognise treatment failure (3–6 months) may provide further opportunity for metastatic spread, before secondary treatment (salvage cystectomy) is undertaken. Also, the morbidity in patients undergoing salvage cystectomy has been found to be significantly higher than in patients undergoing primary cystectomy (Rosario *et al*, 2000). Consequently, if a patient's bladder tumour RT response could be predicted in advance, RT could be promoted in patients with tumours that are predicted to respond. Conversely, patients with nonresponsive tumours could be identified and offered surgery at an earlier stage. In this way, the overall local control rates could be improved.

While much work has been undertaken to develop assays capable of predicting tumour response to RT, none has been successfully applied to clinical practice. However, *ex vivo* measures of the surviving fraction of tumour cells at 2 Gy (SF2) suggest that intrinsic radiosensitivity (IRS) is a significant factor in determining tumour radiocurability (West, 1995; West *et al*, 1997). However, the SF2 assay fails to provide information on a time scale appropriate for treatment planning and also suffers from limited success rates; in an unpublished study, <10% of bladder tumours gave rise to colonies on soft agar (McKeown, McKelvey-Martin and Ho, personal communication). In addition, the relationship between clonogenic survival and clinical response is far from proven. The limitations of SF2 have stimulated research into methods to provide a more rapid and complete measure of IRS.

DNA is the most important cellular target for the lethal effects of ionising radiation, with double-strand breaks (DSBs) proposed to be the principal lesions responsible for radiogenic cell killing (Ward, 1988; Iliakis, 1991). Unfortunately, the relative yield of radiogenic DSBs is low, and high radiation doses tend to be required to produce measurable levels; doses that are far greater than those used clinically. Indeed, while a recent study using the neutral comet assay to measure DSBs reports an association between DSB manifestation and survival (Price *et al*, 2000), the correlations were not definite, with one cell line yielding a false impression of radiosensitivity at the high doses (30 Gy) required for the assay. This highlights a benefit of conducting predictive tests of radiosensitivity at clinically relevant doses.

In contrast with DSBs, the yield of radiation-induced single-strand breaks (SSBs) is far greater (Ward, 1988), and can be readily measured at low clinically relevant doses of radiation. Furthermore, the mechanisms that are proposed to vary the yield of radiation-induced DSBs formation are also expected to vary the yield of radiation-induced SSBs (Ward, 1990). Consequently, the extent of radiation-induced SSB formation can be considered a valid surrogate marker of radiogenic DSB formation.

The alkaline comet assay (ACA) is a highly sensitive method for the assessment of SSBs and alkali labile sites (ALSs), and can readily detect levels of damage induced by clinically relevant doses of radiation (Singh *et al*, 1994; Singh, 1996). In a previous study of just three bladder cancer cell lines, an inverse correlation was obtained between clonogenic survival and mean tail moment (TM) for comet formation, suggesting that ACA could potentially be used to predict the radio response of the single cell lines (McKelvey-Martin *et al*, 1998).

In the present study, we report our evaluation of ACA as a measure of bladder cancer cell radiosensitivity *in vitro* using a panel of six bladder cancer cell lines, and demonstrate that the extent of comet formation best reflects bladder cancer cell radiosensitivity; these results are supported by two independent, parallel studies using colorectal tumour cells (Dunne *et al*, 2003) and bladder tumour cells (McKeown *et al*, 2003). We also report on a preliminary study to determine the differing ACA radio response of epithelial cells isolated from human bladder tumour biopsies.

## MATERIALS AND METHODS

### Cell lines and culture conditions

The six bladder cell lines used in this study (RT112, UM-UC-3, HT1376, J82, T24 and RT4; derived from high-grade TCCs) were purchased from American Tissue Culture Collection (Manassas, USA) and the European Collection of Cell Cultures (Salisbury, UK). All cells were cultured as monolayers in exponential growth by subcultivation. The RT112 and HT1376 cells were cultured in Eagle's minimal essential medium (EMEM), with 10% foetal calf serum (FCS), 1% nonessential amino acid and 1% penicillin–streptomycin. For the J82 cell line, 1% sodium pyruvate and 1% glutamax were also added. The UM-UC-3 cell line was cultured in EMEM with 10% FCS, 1% penicillin–streptomycin and 1% sodium pyruvate. The T24 cell line was cultured in McCoy's medium with 10% foetal calf serum and 1% penicillin–streptomycin. The RT4 cell line was subcultivated in RPMI medium with 10% foetal calf serum, 1% penicillin–streptomycin and 1% sodium pyruvate. All the cell lines used in the present study were tested for mycoplasma contamination and were determined to be free of contamination (‘Mycoplasma Experience’, Reigate, Surrey, UK). Cells were harvested at 70–80% confluence by washing with prewarmed 37°C phosphate-buffered saline (PBS) and then trypsinisation (0.1% trypsin, 0.4% EDTA in PBS) at 37°C. Culture medium was added to the cells before centrifugation at 1500 r.p.m. for 5 min. The pellet was resuspended and incubated in culture medium (37°C, 30 min) at 1 × 10^5^ cells ml^−1^ (for ACA and clonogenic assay) before use. All cultures were tested for viability following this procedure by trypan blue exclusion and was consistently found to be >99%.

### Alkaline comet assay

#### ‘Cell-slide’ preparation

Radiation-induced SSB and ALS DNA damage were assessed using a modified version of ACA, whereby the cells were irradiated ‘set’ in agarose gels on microscope slides (‘cell-slides’). This modified version of the comet assay increases sensitivity by minimising the opportunity for repair of induced damage prior to cell lysis (McKeown *et al*, 2003).

The ACA technique used was adapted from the initial protocol of Singh *et al* (1988). Briefly, dakin-frosted slides were covered with 100 *μ*l of 0.6% normal melting point agarose (dissolved in Ca- and Mg-free PBS at 45°C), and the agarose was allowed to solidify under a coverslip on ice and then the coverslips were removed. Aliquots (1 ml) of harvested cells containing 1 × 10^5^ cells in culture medium were then centrifuged at 1500 r.p.m. for 5 min. The pellets were resuspended in 80 *μ*l of 0.6% low melting point agarose (dissolved in Ca- and Mg-free PBS at 37°C), layered onto the normal melting point agarose and allowed to solidify under a fresh coverslip on ice. For ‘repair-slides’ equal volumes (40 *μ*l) of cell suspension (in RPMI medium) and 1.2% of low melting point agarose (dissolved in RPMI medium containing 20% FCS held at 37°C) were mixed, layered and allowed to solidify under a fresh coverslip on ice. All the steps described were conducted under a reduced light level to prevent additional DNA damage.

#### X-ray irradiations

Cell-slides were irradiated on ice, using a Pantak DXT300 X-ray machine (Radiotherapy Unit, Leicester Royal Infirmary) operated at 300 kVp (HVL of 3.5 mm Cu). Up to 12 slides are placed flat, in a prescribed manner, on an aluminium sheet in thermal contact with ice, 0.8 cm from a 50 cm FSD, 20 cm square, normal therapy applicator. The dose rate (0.98 Gy min^−1^) and uniformity of the field (±10%) were previously determined for this configuration using thermoluminescent dosimeters (not presented). Duplicate or triplicate slides were irradiated with a dose of 2, 4 and 6 Gy to generate an immediate damage dose response. Additional duplicate or triplicate slides irradiated with 2.5 Gy were used to monitor repair at 15 and 30 min.

#### Lysis and electrophoresis

For studies of immediate damage, slides were irradiated and then placed immediately in cold lysis buffer (2.5 M NaCl, 100 mM Na_2_EDTA, 10 mM Tris, pH 10 and 1% Triton X-100 (added fresh), 4°C) for a minimum of 1 h. For repair studies, after irradiation the ‘repair-slides’ were incubated in growth media at 37°C for 15 or 30 min and then placed in lysis buffer. After lysis, the slides were drained and placed in a horizontal gel electrophoresis tank, surrounded by ice and filled with fresh cold electrophoresis buffer (300 mM NaOH, 1 mM Na_2_EDTA, pH 13) to a level of ∼0.25 cm above the slides. Slides were kept in the high pH buffer for 20 min, to allow DNA unwinding. Electrophoresis was then carried out for 20 min at 25 V and 300 mA. The slides were then drained and flooded slowly with three changes of neutralisation buffer (0.4 M Tris, pH 7.5) for 5 min each and then stained with 50 *μ*l of ethidium bromide (20 *μ*g ml^−1^) and covered with a coverslip for immediate analysis.

#### Comet image capture and analysis

A total of 50 cells per slide were analysed to give a representative result for the population of cells (Price *et al*, 2000). Comet image capture and analysis utilised Komet software (Version 4, Kinetic Imaging Ltd, Bromborough, UK) and an epifluorescence microscope (Olympus BH2) fitted with an excitation filter of 515–535 nm, a barrier filter of 590 nm and a 100 W mercury lamp, and operated at a magnification of × 200. The olive tail moment (OTM) was selected as the parameter that best reflected DNA damage (defined as the fraction of tail DNA (TDNA) multiplied by the distance between the profile centres of gravity for DNA in the head and tail). OTM was measured from three independent experiments, each containing duplicate or triplicate measures and presented as the mean±s.e.

### Clonogenic assay

Cells from exponentially growing culture were seeded in appropriate numbers in 60 × 15 mm^2^ Petri dishes with 10 ml of appropriate culture medium. Following 4 h incubation at 37°C, cells were irradiated in the dishes with 2, 4 and 6 Gy. Non-irradiated cultures were processed in parallel. Dishes were subsequently incubated at 37°C in humidified 95% air, 5% CO_2_ atmosphere for 2–3 weeks. Cells were then fixed with 3 : 1 ethanol : acetic acid and stained with crystal violet. Colonies were counted for the control and dose groups, and each experiment was performed on at least three separate occasions in triplicate. The SF, presented as the mean±s.e., was defined as the ratio of colonies formed to cells plated (with correction for plating efficiency (PE)), and calculated using the formula: SF=colonies counted/(cells seeded × (PE/100)). PE was expressed as the number of colonies scored as a percentage of the number of viable control cells plated. This was >35% for all the cell lines.

### Human bladder samples

Samples of human bladder cancer tissue were acquired from eight patients undergoing transuretheral resection for suspected muscle-invasive bladder cancer. Muscle invasion was confirmed by subsequent histological analysis. Acquisition of tissue specimens was approved by the local ethical committee. Samples were transported to the laboratory in culture media (10 ml) immediately following resection. Only exophytic areas of the tumour were sampled, avoiding subepithelial layers of the bladder wall. The tumour material was finely chopped and placed in 20 ml of collagenase (1 mg ml^−1^) in a shaking water bath at 37°C for 20 min. The sample was then filtered through a nylon mesh (120-gauge filter) and the filtrate was washed three times with PBS. The resulting single cells were counted and the viability was assessed by trypan blue exclusion. (typically this was >80%; samples with lower levels of viability were discarded).

To reduce infiltrating cells contaminating the cell suspension, cells expressing human epithelial antigen (HEA) were selected via a biomagnetic technique using HEA microbeads according to the supplier's instructions (Miltenyi Biotech, Bisley, UK). HEA is widely expressed on cells of epithelial origin, including associated tumour cells (Moldenhauer *et al*, 1987).

To account for variation between experiments, additional slides were prepared using an established lymphoblastoid cell line (Raji) and these were irradiated and analysed in parallel.

## RESULTS

Figure 1A

**Figure 1:**
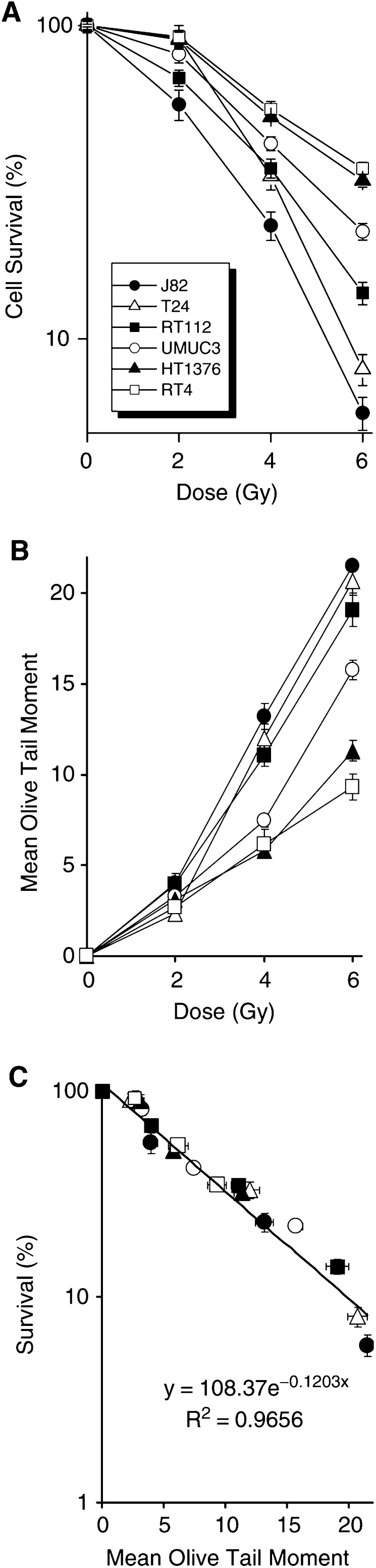
(**A**) The radiation cell survival curve responses for the six bladder cancer cell lines investigated, over a dose range of 0–6 Gy, as determined by clonogenic assay. Survival was determined as the number of colonies formed following X-ray exposure. (**B**) The extent of initial comet formation, as measured by mean OTM, for the six bladder cancer cell lines, over a dose range of 0–6 Gy, as determined by ACA. For (**A** and **B**) each data point is the mean of three independent experiments±s.e. (**C**) The relationship between the measures of mean OTM for initial comet formation, as measured by ACA, and the measures of clonogenic cell survival, for all six bladder cancer cell lines at 0, 2, 4 and 6 Gy. The data are fitted with an exponential trend line and the slope and correlation coefficient are deduced.

shows the radiation cell survival responses for the six bladder cancer cell lines investigated, over a dose range of 0–6 Gy, as determined by clonogenic assay. The determined SF2 values are as follows: RT4 0.92±0.08; T24 0.91±0.054; HT1376 0.9±0.052; UM-UC-3 0.81±0.051; RT112 0.68±0.04; J82 0.56±0.062. The studied cell lines exhibit a range of radiogenic sensitivities, with J82 being the most sensitive to radiation-induced cell killing, and RT4 and HT1376 being the most resistant. Notably, one cell line, T24, by virtue of its unusual dose–response curve, with a prominent shoulder and steep exponential, demonstrates relative radioresistance at a low dose (2 Gy) and relative radiosensitivity at higher doses.

Figure 1B compares the measures of initial comet formation (as assessed by mean OTM) for the six bladder cancer cell lines, over a dose range of 0–6 Gy, as determined by ACA. The plots reveal clear dose–response curves for each cell line, with the most radiosensitive cell line (J82) displaying the highest values (at 6 Gy: OTM 21.5; TM 67.5; % tail DNA (%TDNA) 69%), and the most radioresistant cell line (RT4) the lowest (at 6 Gy: OTM 9.3; TM 19.0; %TDNA 25.5). At 6 Gy, the rank order for initial comet formation matches the rank order of cell killing for all six cell lines (J82>T24>RT112>UM-UC-3>HT1376>RT4). Furthermore, the T24 cell line exhibits a relatively low measure of mean OTM at a low dose (2 Gy), but relatively greater measures at higher doses (4 and 6 Gy). Using the ACA method as described, at 2 Gy there is a significant difference for the measures of mean OTM between the more radiosensitive (J82 and RT112) and radioresistant (T24 and RT4) cell lines (*P*<0.05, Tukey one-way ANOVA).

The relationship between mean OTM for initial comet formation and clonogenic cell survival is shown in Figure 1C. The high degree of correlation between the collated measures for all six cell lines (*R*^2^=0.9656) indicates that the measures of initial comet formation accurately reflects clonogenic survival for these six cell lines over the dose range studied. For each individual cell line, the determined slopes and correlation coefficients (*R*^2^) for comet formation *vs* survival are as follows: RT4 slope, −0.118 (*R*^2^ 0.9575); T24 slope, −0.1226 (*R*^2^ 0.9785); HT1376 slope, −0.1074 (*R*^2^ 0.9485); UM-UC-3 slope, −0.0999 (*R*^2^ 0.9785); RT112 slope, −0.1023 (*R*^2^ 0.9977); J82 slope, −0.1275 (*R*^2^ 0.9867), with the values of the derived individual slopes varying by no more than ∼15% of the collated value (−0.1203).

Figure 2A

**Figure 2:**
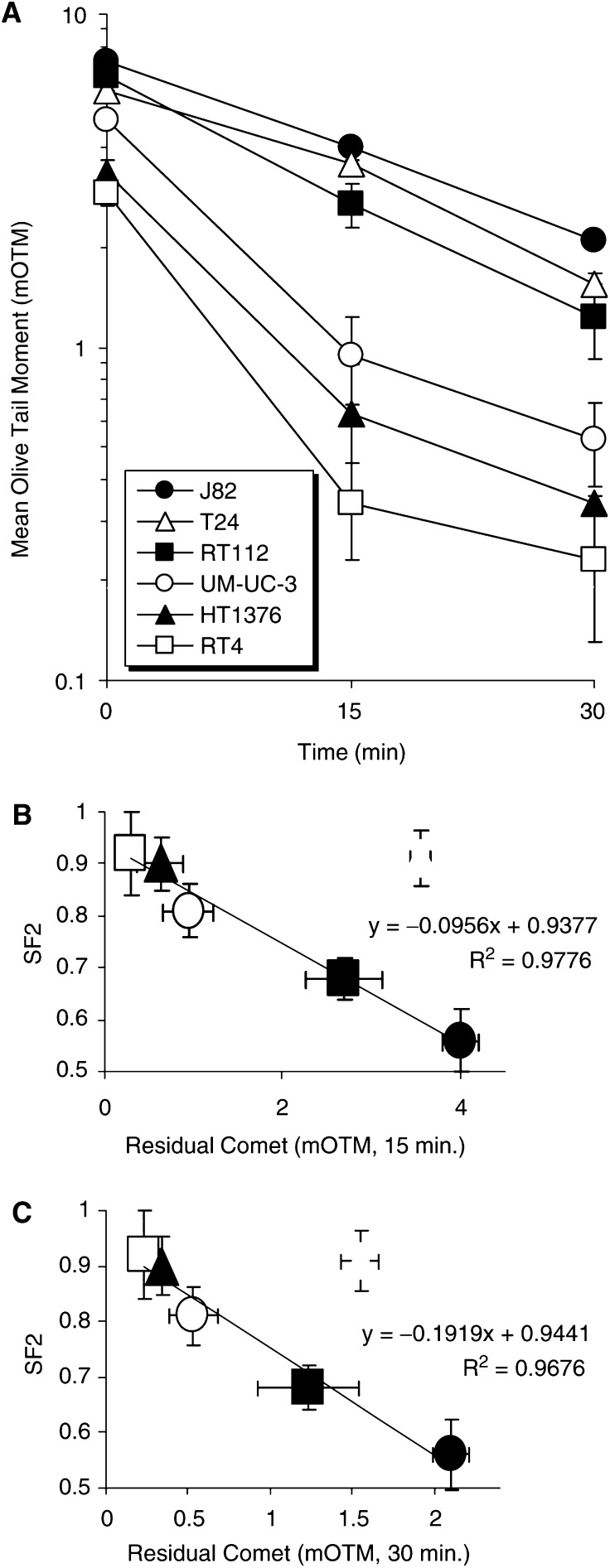
(**A**) The extent of damage repair as determined by ACA, after irradiation with 2.5 Gy of X-rays. Each data point is the mean or three independent experiments±s.e. The dashed line represents the mean ‘background’ level for unirradiated/control cells. (**B** and **C**) The correlation between the measures of residual damage at (**B**) 15 and (**C**) 30 min, as determined by ACA, and the determined SF2 values for the five cell lines J82, RT112, UM-UC-3, HT1376 and RT4. Data points for T24 (open grey triangles) were excluded from the analysis.

illustrates the ACA assessment of damage repair as determined by measurement of residual comets after incubation (37°C) of the ‘cell-slides’ (see Materials and Methods) for either 15 or 30 min, after 2.5 Gy irradiation. For the most radioresistant cell lines (RT4 and HT1376), the majority of repair occurs by 15 min, while for the more radiosensitive cell lines (J82 and RT112), a significant ACA measure of residual comet remains after 30 min. Figure 2B and C show the relationship between the measures of residual comets at the two repair incubation time points (15 and 30 min), as determined by ACA, and the determined SF2 values for the five cell lines J82, RT112, UM-UC-3, HT1376 and RT4. For these five cell lines, the measures of residual comet after 15 and 30 min correlate with SF2 with *R*^2^ values of 0.9776 and 0.9676, respectively. However, the inclusion of the data for the T24 cell line (light grey open triangle) significantly worsened the correlations, yielding *R*^2^ values of 0.39934 and 0.5097 for 15 and 30 min repair, respectively; for the T24 cells, there is a higher ACA measure of residual comet manifest during repair incubation.

Figure 3

**Figure 3:**
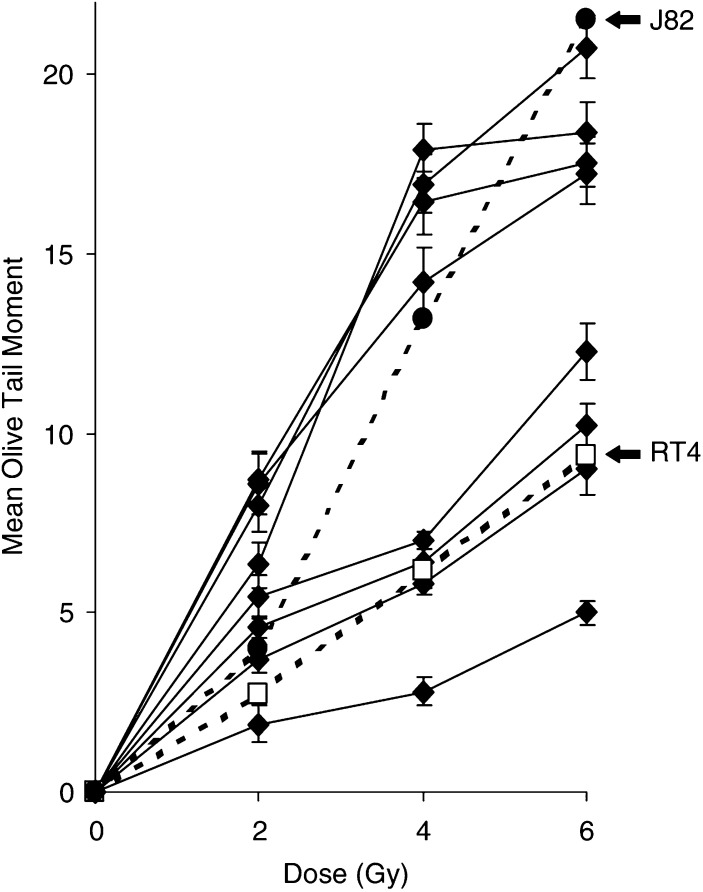
Extent of initial comet formation, as measured by mean OTM, for epithelial cells prepared from invasive bladder tumour biopsies, over a dose range of 0–6 Gy, as determined by ACA. Each data point is the mean of duplicate or triplicate irradiations±s.e. Also shown is the extent of initial comet formation for the bladder cancer cell lines J82 and RT4.

compares the ACA measures of initial comet formation in a pilot study of epithelial cells prepared from biopsies of human invasive bladder tumours (*n*=8), over a dose range of 0 to –6 Gy. The plots of the mean OTM reveal clear dose–response curves, with a >4-fold difference in the initial comet formation being noted between the samples with the highest and lowest measures of comet formation.

## DISCUSSION

The development of rapid and valid predictive tests of cell radiosensitivity has important implications for clinical practice; allowing for the rational choice and tailoring of treatments to each individual patient, based on that individual's tissue–cell radiation sensitivity (Peters and McKay, 2001). The comet assay is a straightforward and highly sensitive method for assessing DNA damage formation and repair at the individual cell level (Singh *et al*, 1988; Singh, 1996), and is particularly suitable for the measurement of radiogenic damage (Olive, 1999). The assay is inexpensive, data are available within 24 h and only low numbers of cells are required, allowing results to be generated from small clinical biopsies. This technique is therefore ideal for clinical application.

In the present study, we demonstrate ACA to be capable of predicting radiation cell survival for a panel of six bladder cancer cell lines. For initial measures of comet formation, there is a strong inverse correlation with clonogenic survival for all six cell lines, with the most radiation-sensitive cell lines displaying the highest measures and the most radioresistant cell lines the lowest. Furthermore, for one cell line, T24, the notable relative change in survival response, from being relatively radiation resistant to relatively radiation sensitive with increasing dose, was mirrored in the ACA response. For the studies of repair, which entailed measures of residual comets at various repair time points, there was good correlation with survival (SF2) for five of the six cell lines investigated, with the apparent extent of repair being greater in the radioresistant cell lines. However, the superior correlation of clonogenic radiation survival with the extent of immediate comet formation, for *all* six cell lines (including the anomalous behaviour of T24), signifies that it is immediate comet formation that best predicts survival for these cell lines. Virtually identical findings are reported in the two accompanying articles (Dunne *et al*, 2003; McKeown *et al*, 2003).

For each individual cell line, the measures of mean OTM increase with dose due to the dose dependency of radiation-induced DNA damage formation. However, a conclusion that it is the differing levels of immediate radiogenic DNA damage noted between cells, which is responsible for their differing radiosensitivities, conflicts with previous reports that fail to detect such damage (SSB or DSB) differences/correlations using comet or other assays (Olive *et al*, 1991; Evans *et al*, 1993; Woudstra *et al*, 1996). This may reflect the fact that studies that fail to correlate initial damage or repair with cell survival tend to have compared cell lines of different origins (Olive *et al*, 1991, 1994; Woudstra *et al*, 1996) which may have different DNA conformations and therefore may exhibit considerable differences in radiogenic DNA damage assays. However, studies using cell lines of the same origin at least show correlations between survival and repair/residual damage (Marples *et al*, 1998; Eastham *et al*, 1999). A further factor that may influence the extent of comet formation is the nature of the DNA anchoring matrix-associated regions (MARs). MARs allow the maintenance of contiguous looped regions of nuclear DNA of varying superhelical densities. Consequently, differences in the character of MARs may result in weaker DNA–matrix associations, thereby allowing additional release of the adjacent/contiguous loops of DNA resulting from the presence of radiation-induced DNA damage. Indeed, studies of radiation-sensitive and -resistant mutants have revealed differences in IRS to correlate with DNA stability in the presence of DNA damage as measured by DNA loop rewinding ability, and with changes in nucleoid protein composition (Malyapa *et al*, 1994, 1996).

In the repair studies, it was noted that residual damage (as assessed by measures of residual comets) correlate well with SF2 for five of the six cell lines, with repair being greater in the radioresistant cell lines. The high degree of correlation between the measures of residual comet and SF2 may reflect the inclusion and contribution of the SSB components of DSBs (and other complex lesions) in the ACA assessment of damage at the time points studied. Many studies have shown that a greater abundance of residual DSBs, after a period of repair, is associated with a higher cellular radiosensitivity, for example (Zhou *et al*, 1998). However, for one cell line (T24), measures of residual comet did not correlate with SF2, there being a greater than expected level of residual damage manifest during repair incubation for this cell line. This may be due to the relative change in the survival of this cell line, from being relatively radioresistant to relatively radiosensitive, at doses >2 Gy; at higher doses, the T24 cells may suffer proportionally greater levels of damage. Alternatively, the relatively slow repair of damage (SSB and ALS) may reflect a possible deficiency in the base excision repair for T24 cells. Obviously, this scenario would seriously confound attempts to predict radiosensitivity from estimates of repair determined by ACA. However, the fact that the unusual survival curve response of T24 was also reflected in the ACA response strongly supports the ability of one assay (ACA) to predict the other (survival). Furthermore, this relative change in survival highlights the benefit of conducting predictive tests using clinically relevant doses of radiation. In this particular system, the use of higher test doses (>2 Gy) would yield a false impression of radiosensitivity for T24 at a low dose (2 Gy). However, this does not totally invalidate high-dose experiments.

In a preliminary pilot study, epithelial cells isolated from invasive bladder cancer biopsies also reveal a range of radio responses, as determined by ACA, encompassing the responses noted for the most radiation-sensitive and -resistant cell lines (J82 and RT4, respectively). Most importantly, this demonstrates that the comet assay is possible on cells from biopsy material. From our investigations with cultured bladder cancer cell lines, we speculate that the >4-fold difference in immediate comet formation in the biopsy-derived epithelial cells potentially reflects similar variations in tumour cell radiosensitivity, but the validity of this remains to be substantiated. However, the observation of differing tumour cell radioresponses, in particular the observation of high degrees of possible radioresistance, may be significant as a contributing factor to the current high level of invasive bladder cancer RT failure (Shipley *et al*, 1985; Duncan and Quilty, 1986).

## CONCLUSIONS

In the present study, we have shown that the initial levels of comet formation, as determined by ACA, best predict bladder cancer cell radiosensitivity at low clinically relevant doses of radiation. The measures of repair correlate with cell survival (SF2), but for only five of the six cell lines studied. In a pilot study, epithelial cells isolated from human bladder cancer biopsies reveal a potential range of predicted radiosensitivities as determined by ACA at clinically relevant radiation doses, and we speculate that this variation in radiosensitivity may reflect the widely differing response of bladder tumours to RT *in vivo*. Overall, our studies demonstrate ACA to be a good predictive measure of bladder cancer cell radiosensitivity. Further studies are required to evaluate the ACA method fully as a valid predictive test of bladder tumour cell radiosensitivity in the clinical treatment of invasive disease.
